# Echocardiography accurately predicts pulmonary hypertension in patients with advanced lung disease

**DOI:** 10.1186/s13054-017-1697-y

**Published:** 2017-05-25

**Authors:** Silvia Cottini, Christian Benden, Lars C. Huber, Mattia Arrigo

**Affiliations:** 1 Intensive Care Unit, University Hospital Zurich, Zurich, Switzerland; 2 Division of Pulmonology, University Hospital Zurich, Zurich, Switzerland; 3 Clinic for Internal Medicine, City Hospital Triemli, Zurich, Switzerland; 4 Department of Cardiology, University Hospital Zurich, Raemistrasse 100, 8091 Zurich, Switzerland

**Keywords:** Pulmonary hypertension, Echocardiography, Advanced lung disease, Transplantation, Right heart catheterization

Pulmonary hypertension (PH) may be observed in many different conditions, including advanced heart or lung disease [1]. PH is defined as elevation of the mean pulmonary arterial pressure (mPAP) ≥25 mmHg, measured by right heart catheterization (RHC). Since procedure-related risks and costs are not negligible, RHC is not routinely performed in patients with advanced lung disease during initial assessment and follow-up. Echocardiography is commonly used as screening tool for the presence of PH, but might be of limited diagnostic value, in particular in patients with lung disease [2]. Empirical formulas were proposed to calculate the mPAP starting from systolic pulmonary arterial pressure (sPAP) as assessed by echocardiography [3, 4]. It has been suggested that both the Chemla formula (mPAP = sPAP × 0.61 + 2 mmHg) and the Syyed formula (mPAP = sPAP × 0.65 + 0.55 mmHg) might accurately estimate the mPAP [5]. The aim of our study was to assess the accuracy of these echocardiography-based formulas to estimate the invasively measured mPAP in a cohort of patients with advanced lung disease. Estimation of sPAP by echocardiography was performed according to current recommendation by adding to the right ventricular/right atrial pressure gradient (based on Doppler-measured tricuspid regurgitation velocity) the estimated right atrial pressure (based on inferior vena cava diameter and collapsibility). A total of 96 consecutive patients undergoing lung transplant evaluation between 03/2000 and 10/2015 were included. Data from RHC and echocardiography performed the same day were available for the whole cohort.

Spearman’s rank-order correlation was run to assess the relationship between RHC-measured mPAP and calculated mPAP. There was a strong positive correlation between both RHC-mPAP and Chemla-mPAP (rs = 0.909, *p* < 0.001; Fig. 1a) and between RHC-mPAP and Syyed-mPAP (rs = 0.904, *p* < 0.001; Fig. 1b).

**Fig. 1:**
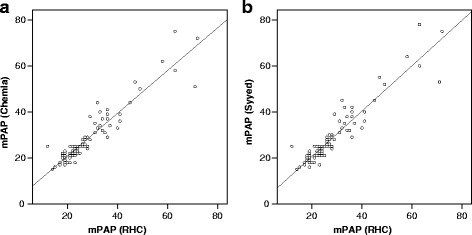
Correlation between RHC-measured mPAP and echocardiographic-estimated mPAP using the Chemla formula (**a**) and the Syyed formula (**b**). Values expressed as mmHg

Inspection of both diagrams suggests that the correlation between echocardiographic data and invasive pulmonary hemodynamics is of particular strength in patients with moderate elevation of pulmonary pressure. Whether echocardiography-based formulas are of similar accuracy to estimate pulmonary pressure of PH in patients with severe PH remains unclear. However, this might be of minor relevance since, as shown in our cohort, only a minority of patients with advanced lung disease present with a mPAP >50 mmHg. In these patients, further invasive assessment of pulmonary hemodynamics may be indicated. For all other patients our data emphasize that echocardiography is an accurate tool to estimate pulmonary pressure in patients with advanced lung disease.

## Abbreviations

mPAP: Mean pulmonary arterial pressure
PH: Pulmonary hypertension
RHC: Right heart catheterization
sPAP: Systolic pulmonary arterial pressure
